# Causes and predictors of mortality in Asian Indians with and without diabetes–10 year follow-up of the Chennai Urban Rural Epidemiology Study (CURES - 150)

**DOI:** 10.1371/journal.pone.0197376

**Published:** 2018-07-09

**Authors:** Ranjit Mohan Anjana, Ranjit Unnikrishnan, Poongkunran Mugilan, Padoor Sethuraman Jagdish, Balasubramanian Parthasarathy, Mohan Deepa, Geetha Loganathan, Rajendran Ashok Kumar, Thangarajan Rahulashankiruthiyayan, Ganesan Uma Sankari, Ulagamathesan Venkatesan, Viswanathan Mohan, Coimbatore Subramanian Shanthi Rani

**Affiliations:** Madras Diabetes Research Foundation & Dr. Mohan’s Diabetes Specialities Centre, WHO Collaborating Centre for Non-communicable Diseases Prevention and Control &ICMR Center for Advanced Research on Diabetes, Chennai, India; Florida International University Herbert Wertheim College of Medicine, UNITED STATES

## Abstract

**Background:**

The incidence and prevalence of diabetes is increasing worldwide and it is the fifth leading cause of mortality accounting for over 3.8 million deaths annually. Despite the enormity of the diabetes-related health burdens, very few studies have evaluated the factors associated with mortality among people with diabetes in India. We sought to study the causes and predictors of mortality among urban Asian Indians with and without diabetes.

**Methods and findings:**

Of 2273 adults (27,850 person-years of follow-up) from the 10-year follow-up of the Chennai Urban Rural Epidemiology Study (CURES), the cause of death could be ascertained in 552 individuals out of the 671 who had died (response rate 82.3%). Verbal autopsy was obtained from the family members of the deceased and this was adjudicated by trained physicians. The age-standardized mortality rate was 28.2 (95%CI 25.9–30.6) per 100,000 population. Mortality rates were significantly higher in individuals with diabetes compared to those without [27.9(95% CI 25.5–30.6) vs. 8.0 (6.6–9.9) per 1000 person years]. Compared to individuals of normal body mass index, underweight individuals had higher risk of mortality (Hazard ratio 1.49; 95% CI 1.11–2.0), whereas overweight and obese individuals did not show a higher risk. The population-attributable risk for all-cause mortality in the entire study cohort was highest for ischemic heart disease and diabetes. The excess mortality attributable to diabetes was highest in the age group of 51 to 70 years, and was mostly accounted for by renal disease (Rate ratio 5.68, 95%CI 2.43–6.23), ischemic heart disease (4.23,2.78–6.67), and cerebrovascular disease (4.00,1.87–9.81).

**Conclusion:**

Underweight (but not overweight or obesity) was strongly associated with mortality in this Asian Indian population. Ischemic heart disease and diabetes contributed the most to risk for all cause mortality. Excess mortality due to diabetes was higher in relatively younger individuals and was mostly accounted for by renal disease.

## Introduction

The incidence and prevalence of diabetes is increasing worldwide, with the prevalence estimated to almost double by 2030 [1]. Diabetes is the fifth leading cause of mortality accounting for over 3.8 million deaths annually [2]. It has been estimated that one individual dies of diabetes or its complications every 10 seconds. Most of the excess mortality due to diabetes is attributable to cardiovascular disease (CVD) and renal failure [3]. Individuals with diabetes have an increased risk of all-cause mortality and morbidity related to CVD compared with individuals without diabetes [4–5].

South Asia is one of the epicenters of the diabetes pandemic. Asian Indians with diabetes have been shown to have higher mortality rates compared to other ethnic groups [6]. Despite the enormity of the diabetes-related health burdens, very few studies have evaluated the factors associated with mortality among people with diabetes in India.

In the present study, we attempt to estimate the mortality rates, the causes of death among individuals with and without diabetes, the excess risk of death associated with diabetes and also the population attributable risk (PAR) for death associated with various modifiable risk factors in the follow-up cohort of a large epidemiological survey, conducted on a representative population of Chennai, the largest city in South India.

## Research design and methods

### Baseline studies

**Fig 1:**
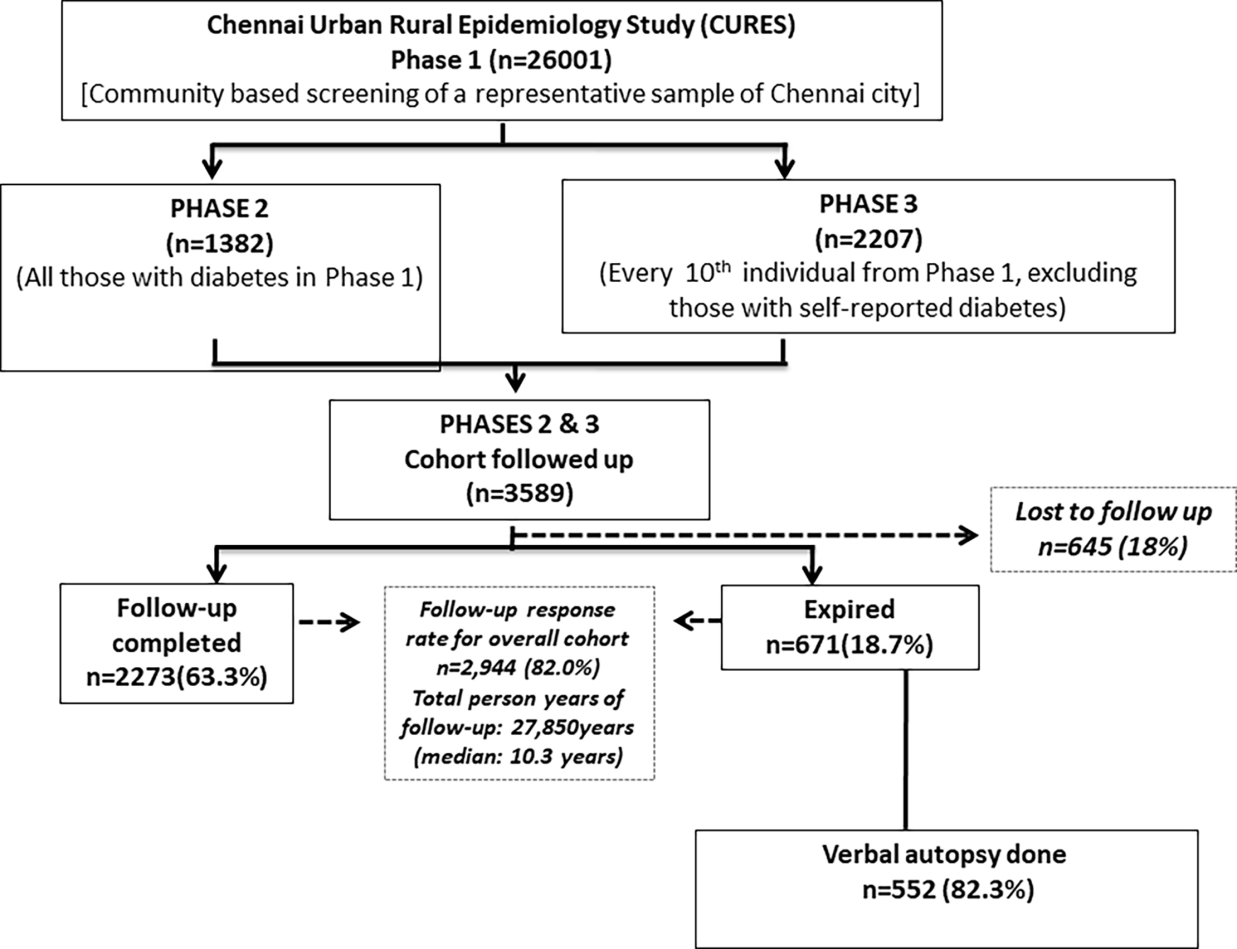
Flow chart of the study cohort.

The present paper reports on the mortality rates in the 10-year follow-up of the Chennai Urban Rural Epidemiology Study (CURES) cohort. The methodology of CURES has been published elsewhere [7]. In brief, CURES was done on a representative sample of 26001 adults from Chennai city, aged ≥20 years. In the baseline survey carried out between 2001 and 2003, all 26001 individuals were screened for diabetes. Of these, all individuals who had diabetes (n = 1382) and one in every tenth individual (n = 2207) underwent further detailed anthropometric and biochemical investigations and these constituted the cohort for this follow-up study (n = 3589) [8] [**Fig 1**]. Madras Diabetes Research Foundation Institutional Ethics Committee approval was obtained, and written informed consent to use anonymized medical data was obtained from all study subjects before the start of the study. There were no minors in the study.

### Anthropometric and biochemical assessment

At baseline, details pertaining to demography, medical history, family history, tobacco and alcohol use and physical activity were elicited using a structured, pretested and validated interviewer-administered questionnaire. Smokers were defined as those who were currently smoking and alcohol use was defined as current alcohol consumption. Anthropometric details (such as height, weight, waist circumference, hip circumference) and blood pressure were measured using standardized techniques and body-mass index (BMI) calculated as weight in kilograms divided by height in meters squared. A venous blood sample was drawn in the fasting state and 2 hours after oral administration of 75 g of glucose in all individuals who did not give a history of diabetes. Biochemical analyses were done in a laboratory certified by the National Accreditation Board for testing and calibration Laboratories (NABL) and the College of American Pathologists (CAP) on a Hitachi—912 Autoanalyser (Hitachi, Germany) using kits supplied by Roche Diagnostics (Basel, Switzerland), for estimation of plasma glucose (GOD-POD method), serum cholesterol (CHODPAP method), serum triglycerides (GPO-PAP method) and HDL cholesterol (direct method). Glycated hemoglobin (HbA1C) was estimated by high pressure liquid chromatography using the Variant machine (Bio-Rad, Hercules, Calif., USA). The intra and inter-observer coefficients of variation for the biochemical assays ranged from 3.1 to 7.6%.

## Definitions

Diabetes was diagnosed, if the venous plasma glucose 2 hours after oral glucose load (2-hr plasma glucose [2Hr PG]) was ≥200 mg/dl (11·1 mmol/l) and/or the fasting plasma glucose (FPG) levels were ≥126 mg/dl (7·0 mmol/l) [9]

Underweight was defined as a BMI <18.5 kg/m^2^ for both genders (based on the Asia Pacific Guidelines and World Health Organisation criteria) [10,11].

Overweight was defined as a BMI ≥23 but <25 kg /m^2^ (based on the Asia Pacific Guidelines) and ≥ 25 but < 30 kg /m^2^ for both genders (based on the WHO criteria) [10, 11].

Obesity was defined as a BMI ≥ 25 kg/m^2^(Asia Pacific Guidelines) and >30 kg/m^2^ (WHO criteria) for both genders [10,11].

Grade I obesity was defined as a BMI ≥ 25 but < 30 kg/m^2^ (Asia Pacific Guidelines) and ≥ 30 but < 35 kg/m^2^ (WHO criteria) [10, 11].

Grade II obesity was defined as a BMI >30 kg/m^2^ (Asia Pacific Guidelines) and >35 kg/m^2^ (WHO criteria) [10, 11].

Excess mortality was defined according to the World Health Organization criteria in which excess mortality can be expressed as a mortality rate, which is the risk of dying from that condition in a specific population “or” a total number of excess deaths [12].

Assessment of diabetes complications, including retinopathy, microalbuminuria, macroalbuminuria, neuropathy, peripheral vascular disease (PVD) and coronary artery disease (CAD), was performed as described below.

### Retinopathy

A comprehensive ocular examination was performed and visual acuity was recorded using an illuminated Snellen chart. A detailed retinal (fundus) examination using direct and indirect ophthalmoscopy was performed by a retinal specialist trained in grading of retinal lesions. Retinal (fundus) photography was performed using four-field stereo color retinal photography (model FF 450 Pluscamera; Carl Zeiss, Jena, Switzerland) whenever possible. An Early Treatment Diabetic Retinopathy Study grading system that has been modified and standardized in other population-based studies was used for the diagnosis of diabetic retinopathy [13].

### Nephropathy

Urinary albumin concentration was measured in a fasting urine sample using an immunoturbidometric assay (Hitachi 902 autoanalyzer; Roche Diagnostics). Microalbuminuria was diagnosed if the albumin excretion was between 30 and 299 g/mg creatinine. Macroalbuminuria was diagnosed if albumin excretion was ≥300 g/ mg creatinine. Nephropathy was defined as presence of either micro- or macroalbuminuria [14]

### Neuropathy

Neuropathy was assessed using a biothesiometer. Vibratory perception threshold of the great toes was measured in a standardized manner by a single observer, and neuropathy was diagnosed if the mean vibratory perception threshold was ≥20 V [15].

### Peripheral vascular disease

PVD was diagnosed by measurement of ankle-brachial pressure index (ABPI) using a Doppler probe. Blood pressure recordings were made of the brachial pulses in the upper limb. Similar recordings were made of the dorsalis pedis and posterior tibial pulses in the lower limb by inflating the cuff proximal to the ankle, and the mean of these two readings was taken as the ankle pressure. ABPI <0.9 was considered diagnostic of PVD [16].

### Coronary artery disease

CAD was diagnosed based on a documented past history of myocardial infarction or drug treatment for coronary artery disease and/or Minnesota codes 1-1-1 to 1-1-7, [Q wave changes] 4–1 to 4–2 [ST segment depression] or 5–1 to 5–3 [T wave abnormalities] on the ECG [17].

### Follow up data on mortality

The follow-up survey was done in 2012–2014 after a median of 10.3years (27,850 person-years of follow-up). Out of 3589 individuals in the follow-up cohort, 645 individuals were lost to follow-up (18%). This included 636 individuals who had migrated and were not traceable and 9 who refused to participate even after repeated attempts. It was found that 671 individuals had died, in 552 of whom (82.3%) the cause of death could be ascertained. Information on death was obtained from the study participant’s family members. The cause of death was ascertained through medical records, death certificates or discharge summaries from hospitals. Verbal autopsy was used for estimating cause specific mortality in cases without medical death certification. Data on symptoms reported by caregivers along with the cause of death were collected from a medical facility, and the cause-of-death distribution was estimated in the population where only symptom data were available. These documents were adjudicated by trained physicians.

Deaths in the cohort that occurred between 2001 and 2014 were recorded. The primary endpoint for this study was mortality from all causes, CVD, renal failure, cancer and other causes. Causes of death were coded as ischemic heart disease, renal failure, cerebrovascular disease, diabetes, cancer, chronic obstructive lung disease, liver failure, circulatory system diseases, tuberculosis, congestive heart failure, accident/suicide and unspecified causes of death according to the International Classification of Diseases, Tenth Revision (ICD-10).

## Statistical analyses

Mortality rates were calculated by dividing number of deaths by total person-years observed. Person-years were calculated from the baseline examination until the event (death) occurred. Standardized mortality rates were calculated by dividing sum of products (age specific rate per 100,000 population X weight in the standard population.) across all age groups by sum of weight in standard population (World Health Organization) [18] for persons ≥ 20 years. Statistical analysis was carried out using the SPSS PC Windows version 15.0 [Chicago, IL]. Students “t” test was used to compare means and Chi Square or Fischer’s exact test to compare proportions.

To assess risk for all cause mortality, cox proportional hazard model was used. The variables which were found to be significant when taken individually in the model were considered in the multiple cox proportional hazard model for estimating the hazard ratio and 95% confidence intervals.

Partial PAR and 95% confidence intervals for single modifiable factors adjusted for confounders were estimated for the overall cohort using the method described by Spiegelman [19]. PAR is said to be partial when one or more risk factors are considered to be eliminated while others are allowed to remain unchanged. In our analysis, the fixed factors included age and gender. The following formula [17] was used to calculate the PAR_p_.

$$PARp=\frac{\sum_{s=1}^{S}\sum_{t=1}^{T}p_{st}RR_{1s}RR_{2t}-\sum_{s=1}^{S}\sum_{t=1}^{T}p_{st}RR_{2t}}{\sum_{s=1}^{S}\sum_{t=1}^{T}p_{st}RR_{1s}RR_{2t}}=1-\frac{\sum_{t=1}^{T}p_{.t}RR_{2t}}{\sum_{s=1}^{S}\sum_{t=1}^{T}p_{st}RR_{1s}RR_{2t}}$$

In the formula mentioned above, t denotes a stratum of distinct combinations of levels of all background risk factor (t = 1, 2, 3,…,T) that are not considered in the study whereas s indicates an index exposure group defined by each of the unique combinations of the levels of the index risk factors for which the PAR_p_ applies (s = 1,2, 3,…,S). RR_1s_ is the relative risk analogous to combinations relative to the lowest risk combination, RR_1,1_ = 1. The combined prevalence of exposure group s and stratum t is designated by P_st_ and *p_st_* and $p_{.t}=\sum_{s}^{s}=1$ [19].

## Results

The overall all-cause mortality rate was 19.8per 1000 person years [95% Confidence Interval (CI) 18.2–21.5].The age standardized mortality rate was 28.2 (95%CI 25.9–30.6.) per 100,000 population. The death rate was significantly higher among individuals with diabetes compared to those without [27.9 (95% CI 25.5–30.6) vs 8.0 (95% CI 6.6–9.9) per 1000 person years].

The clinical and biochemical parameters of the study population with and without diabetes is shown in Table 1. [both those who were alive at the time of follow up (n = 2276) and those who were deceased (n = 552)]. The mean age at time of death did not differ significantly between individuals with and without diabetes (65.0±11.2 vs. 66.1±16.5 years respectively). Among individuals with diabetes, significantly more females than males died. Baseline BMI, waist circumference, fasting plasma glucose, HbA1c, serum cholesterol and serum triglycerides were significantly higher among deceased individuals with diabetes compared to those without (p<0.001 for all parameters). The mean duration of diabetes at the time of death was 10.7 ± 6.6 years. Smoking and alcohol consumption were significantly more frequent among deceased individuals without diabetes than those with diabetes. Among those who were alive at follow-up, mean age at the time of follow-up, blood pressure. BMI, waist circumference, fasting plasma glucose, HbA1c, serum cholesterol and serum triglyceride levels were significantly higher among those with diabetes. Prevalence of abdominal obesity, hypercholesterolemia and hypertriglyceridemia were higher among both deceased and alive individuals with diabetes compared to those without.

**Table 1:** Baseline characteristics of subjects with and without diabetes among the study cohort.

Variables	Deceased			Alive		
	Individuals without diabetes (n = 92)	Individuals with diabetes (n = 460)	p value	Individuals without diabetes (n = 1032)	Individuals with diabetes (n = 1244)	p value
Age at the time of death (years)	66.1 ± 16.5	65.0 ± 11.2	0.56	–	–	–
Age at the time of follow up (years)	–	–	–	46.6 ± 12.0	55.7 ± 10.8	<0.001
Gender (male) n (%)	54 (58.7)	205 (44.6)	0.03	437 (42.3)	531 (42.7)	0.87
Duration of diabetes at the time of death (years)	–	10.7 ± 6.6	–	–	–	–
Duration of diabetes at the time of follow up (years)	–	–	–	–	8.9 ± 6.4	–
Systolic blood pressure (mm/Hg)	130 ± 25	133 ± 23	0.22	117 ± 17	126 ± 20	<0.001
Diastolic blood pressure (mm/Hg)	78 ± 12	77 ± 12	0.32	73 ± 11	77 ± 11	<0.001
Body mass index(kg/m^2^)	21.6 ± 4.3	24 ± 4.6	<0.001	23.5 ± 4.7	25.6 ± 4.2	<0.001
Waist circumference (cm)	82.0 ± 11.3	87.9 ± 11.4	<0.001	83.0 ± 12.2	90.6 ± 10.0	<0.001
Fasting blood glucose (mg/dl)	86 ± 8	177 ± 88	<0.001	84 ± 8	135 ± 59	<0.001
Glycated haemoglobin (%)	5.7 ± 0.4	9.1 ± 2.6	<0.001	5.5 ± 0.5	7.7 ± 2.1	<0.001
Serum cholesterol (mg/dl)	184 ± 37	205 ± 46	<0.001	175.8 ± 37.7	195 ± 38	<0.001
Serum triglyceride* (mg/dl)	105.5	154.3	<0.001	99.5	144.5	<0.001
Smoking (yes) n(%)	29 (33.0)	90 (20.0)	0.01	165 (16.0)	193 (15.5)	0.76
Alcohol (yes) n(%)	27 (30.3)	88 (19.6)	0.02	210 (20.3)	237 (19.1)	0.44
**Risk Factors**						
Physical inactivity n(%)	78 (87.6)	395 (88.0)	0.930	846 (82.0)	1031 (82.9)	0.57
Abdominal obesity n(%)	490 (47.6)	916 (74.2)	<0.001	34 (37.4)	279 (61.7)	<0.001
Hypertension n(%)	140 (13.6)	355 (28.6)	<0.001	30 (32.6)	190 (41.4)	0.12
Hypercholesterolemia n(%)	232 (22.5)	527 (42.4)	<0.001	25 (27.2)	243 (53.2)	<0.001
Hypertriglyceridemia n(%)	188 (18.2)	558 (44.9)	<0.001	14 (15.2)	234 (51.3)	<0.001

* Geometric mean; data are mean ± SD.

The most frequent causes of mortality among individuals with diabetes were ischemic heart disease [34.3%], renal failure [10.7%] and cerebrovascular disease [10%], while among individuals without diabetes, ischemic heart disease [28.3%], circulatory disease [9.8%] and chronic obstructive lung disease [9.8%], were the most frequently cited causes of death. There were no significant differences in the frequency of various causes of death between the two groups, except for suicide, which was more frequent in individuals without diabetes (Table 2).

**Table 2:** Causes of death in those with and without diabetes.

Causes of death (With ICD codes)	Individuals without diabetes n (%)	Individuals with diabetes n (%)	p value
Ischemic heart disease (I 20—I 52)	26 (28.3)	158 (34.3)	0.26
Renal failure (N 17-N 19)	6 (6.5)	49 (10.7)	0.23
Diabetes (E 10—E 14)	0	43 (9.3)	<0.001
Cancer (C 00—C 96)	7 (7.6)	35 (7.6)	1.00
Circulatory disease (I 00—I 99)	9 (9.8)	33 (7.2)	0.39
Chronic obstructive lung disease(J40-J47)	9 (9.8)	27 (5.9)	0.16
Chronic liver disease (K 70-K 77)	3 (3.3)	13 (2.8)	0.74
Tuberculosis (A 15—A19)	3 (3.3)	9 (2.0)	0.43
Congestive heart failure (I 50)	4 (4.3)	6 (1.3)	0.05
Others–accident (W 00—W 19)	7 (7.6)	16 (3.5)	0.07
Others–suicide (X 60 -X 84)	8 (8.7)	6 (1.3)	<0.001
Unspecified causes of death(R 45—R 99)	2 (2.2)	19 (4.1)	0.55

**Table 3** shows the hazard ratios (HR) associated with various risk factors for all-cause mortality in the overall study cohort. Increasing age [HR1.008, 95% CI 1.007–1.009, p<0.001], smoking [HR 1.338, 95% CI 1.009–1.775, p = 0.04] and higher glycated hemoglobin [HR 1.264, 95% CI 1.223–1.306, p<0.001] were all independent risk factors for all-cause mortality in this cohort. Among individuals with diabetes, additionally gender, abdominal obesity **(Table 4)** and presence of any three or above chronic complications was associated with an increased risk of mortality. When the HR between groups were compared, only gender, smoking and alcohol were found to be significant with an interaction test p value < 0.05.

**Table 3:** Hazard ratios of various risk factors for all-cause mortality in the overall study cohort.

Variables	Hazard Ratio (95%CI)	P value
Age (years)	1.008 (1.007–1.009)	<0.001
Gender (Male)	0.804 (0.644–1.004)	0.05
Hypertension(≥140/90 mmHg or using drug treatment)	1.199 (0.995–1.444)	0.06
Hypercholesterolemia(≥200 mg/dL)	1.111 (0.923–1.338)	0.26
Hypertriglyceridemia (≥150mg/dL)	1.142 (0.946–1.377)	0.17
Smoking (Yes)	1.338 (1.009–1.775)	0.04
Alcohol (Yes)	1.154 (0.870–1.531)	0.32
HbA1c (%)	1.264 (1.223–1.306)	<0.001

**Table 4:** Hazard ratios of various risk factors for all-cause mortality among subjects with and without diabetes.

	Individuals with diabetes		Individuals without diabetes	
Variables	Hazard ratio	p value	Hazard ratio	p value
	(95% CI)		(95% CI)	
			** **	
Age (years)	1.008 (1.007–1.009)	<0.001	1.010 (1.008–1.012)	<0.001
Gender (Male)	0.709 (0.550–0.913)	0.008	1.037 (0.587–1.831)	0.9
Hypertension	1.181 (0.968–1.442)	0.102	1.089 (0.656–1.807)	0.741
(≥140/90 mmHg or using drug treatment)				
Physical inactivity	0.990 (0.740–1.324)	0.945	0.541 (0.283–1.036)	0.064
Abdominal obesity	0.607 (0.491–0.751)	<0.001	0.629 (0.388–1.020)	0.06
HbA1c %)	1.023 (1.018–1.029)	<0.001	1.346 (0.836–2.167)	0.221
Hypercholesterolemia	1.155 (0.946–1.411)	0.156	0.807 (0.472–1.380)	0.433
(≥200 mg/dL)				
Hypertriglyceridemia	1.112 (0.911–1.357)	0.297	0.718 (0.378–1.365)	0.312
(≥150 mg/dL)				
Smoking (Yes)	1.232 (0.900–1.687)	0.194	1.898 (0.957–3.766)	0.067
Alcohol (Yes)	1.107 (0.807–1.518)	0.53	1.357 (0.687–2.679)	0.38
	Complications*^¤^ **in those with diabetes**			
Any one complication	1.123 (0.857–1.471)			0.4
Any two complications	1.106 (0.827–1.479)			0.498
Any three complications	2.236 (1.615–3.094)			<0.001
All four complications	2.605 (1.516–4.475)			<0.001

^*¤^Adjusted for age and gender

^¤^Complication**s include**—retinopathy, nephropathy, neuropathy, peripheral vascular disease and coronary artery disease.

**Fig 2:**
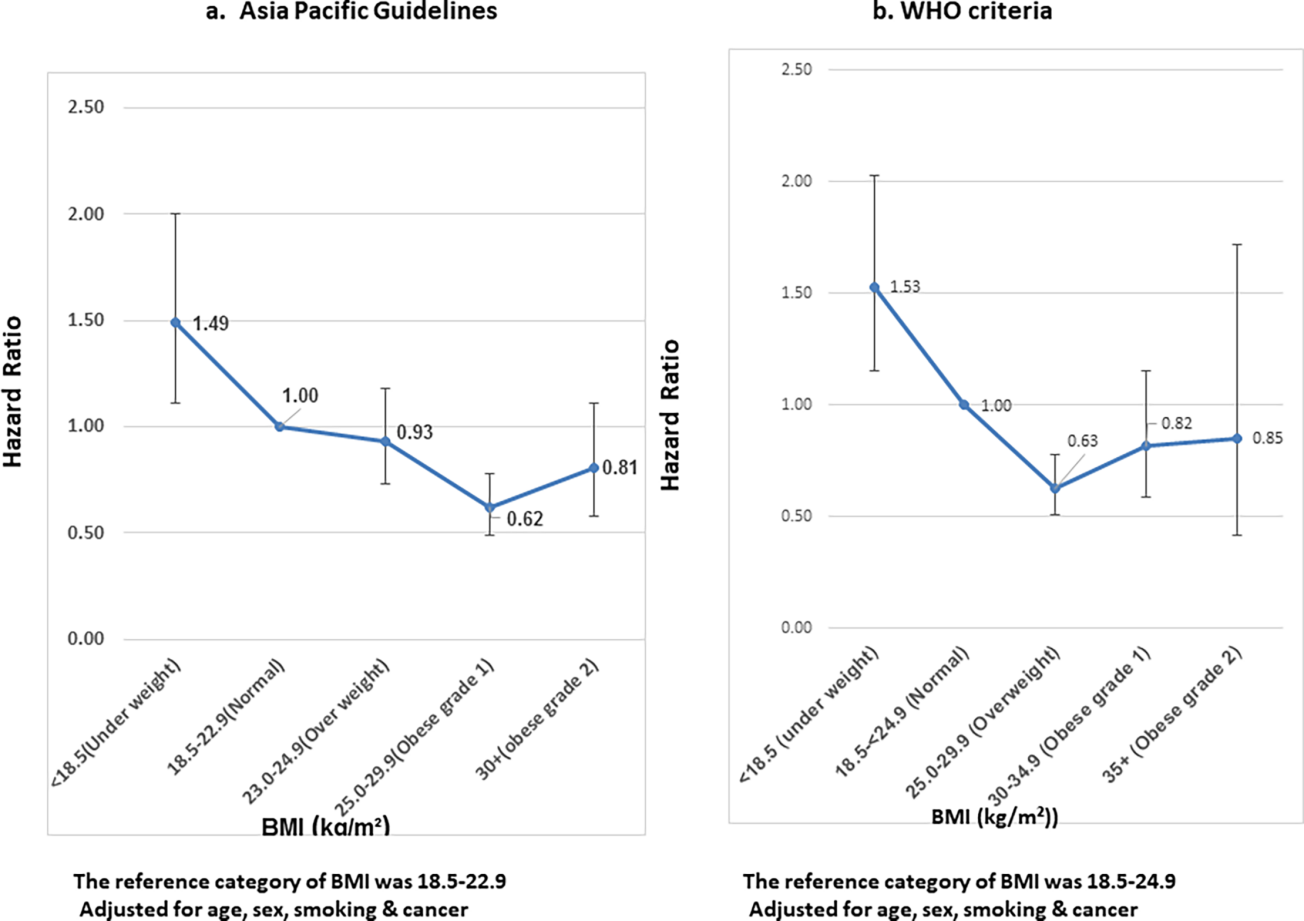
Hazard ratios for all-cause mortality based on BMI.

**Fig 2** show the adjusted hazard ratios for all-cause mortality based on BMI categories (as defined by the Asia Pacific guidelines and WHO criteria) in the overall study population. The risk of death from any cause was higher among individuals with lower-than-normal BMI. Compared to individuals with normal BMI, individuals defined as underweight had a hazard ratio for mortality of 1.49 (95% CI 1.11–2.0) (when the Asia Pacific guidelines were used) and 1.53 (95% CI 1.15–2.03) (based on the WHO criteria). Overweight and obesity were not associated with higher hazard of mortality. The magnitude of the association was similar between the guidelines and was not altered by adjustment for age, gender, cancer and smoking status When split by disease status i.e. diabetes and no diabetes this trend did not change significantly.

**Fig 3:**
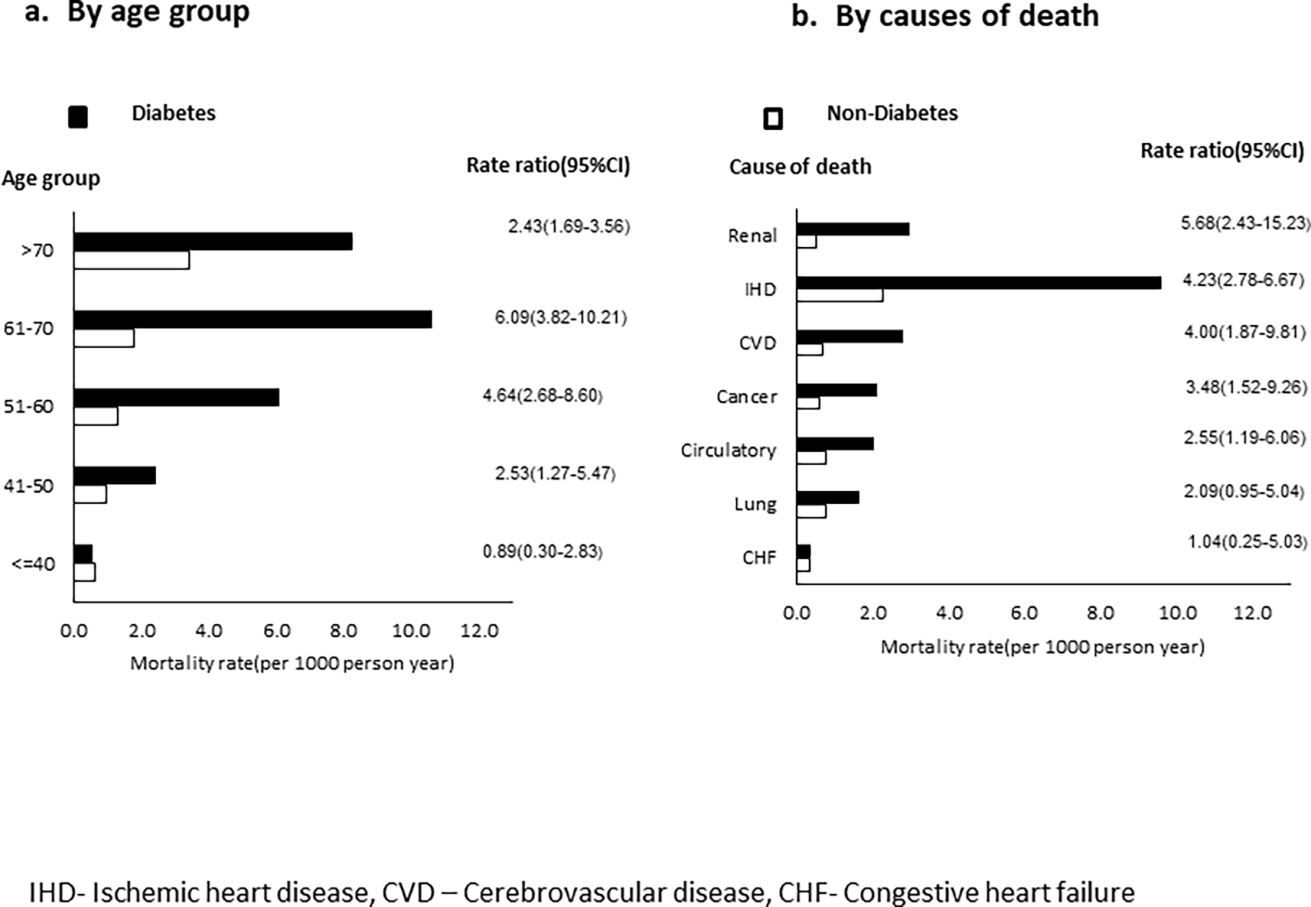
Excess mortality among individuals with and without diabetes.

Excess mortality among individuals with diabetes was highest in the age group of 61–70 years (rate ratio 6.09, 95% CI 3.82–10.21) followed by 51–60 years (4.64, 2.68–8.60) (**Fig 3).** Among the various causes of death, excess mortality among individuals with diabetes was highest for renal disease (rate ratio 5.68, 95% CI 2.43–16.23), followed by ischemic heart disease (4.23, 2.78–6.67) and cerebrovascular disease (4.00, 1.87–9.81)

**Table 5** shows the individual contributions of various modifiable risk factors to the PAR for all cause mortality in the overall study population. Among the modifiable risk factors, physical inactivity was found to contribute most to mortality [PARp 24.7%(4.7–42.8)], followed by hypercholesterolemia, hypertriglyceridemia, smoking, alcohol and least by hypertension [4.1% (7.1–15.2)]. Ischemic heart disease was found to contribute the most to mortality [PARp 53%, (95% CI 25.6–72.5)] followed by diabetes [50.3%, (36.8–61.8)], renal failure [23.6% (4.3–41.1)] and cerebrovascular disease [13.9%(3.4–30.3)].

**Table 5:** Partial population attributable risk for all-cause mortality in the study cohort.

Risk Factors	Adjusted PAR(Partial)*
Physical inactivity	24.7(4.7–42.8)
Hypercholesterolemia	11.9 (2.5–2.1)
Hypertriglyceridemia	11.8 (4.3–19.2)
Smoking	10.2 (2.2–1.8)
Alcohol	9.1 (7.6–10.7)
Hypertension	4.1 (7.1–15.2)
**Causes of Death**	
Ischaemic heart disease	53.0 (25.6–72.5)
Diabetes	50.3 (36.8–61.8)
Renal failure	23.6 (4.3–41.1)
Cerebrovascular disease	13.9 (-3.4–30.3)
Congestive heart failure	4.8 (-10.9–20.2)

## Discussion

To our knowledge, this is the first study from India to compare mortality rates in a representative cohort of individuals with and without diabetes, and to assess the effects of various modifiable risk factors on all-cause mortality. The major findings are as follows: (i) mortality rates are more than three times higher in individuals with diabetes compared to those without diabetes; (ii) underweight was associated with a significantly higher risk of all-cause mortality in the overall study cohort, whereas overweight and obesity were not; (iii) excess mortality due to diabetes was most marked in the age group of 51–70 years; and (iv) most of the excess mortality due to diabetes was accounted for by renal disease, ischemic heart disease and cerebrovascular disease.

While several prospective studies have documented increased mortality associated with diabetes [20–21], earlier studies from India do not provide information on death rates in the general population for comparison [22]. In this study, we report that the mortality rate was almost thrice as high in individuals with diabetes compared to those without (27.9 vs 8.0 per 1000 person-years). This figure is comparable with the results of a previous small study done by our group a decade ago in two selected residential colonies in Chennai, in which the mortality rates were found to be18.9and 5.3 per 1000 person-years among persons with and without diabetes respectively [23]. Studies from China have also shown that individuals with diabetes have a threefold higher death rate compared to age-matched controls without diabetes [24]. Studies from the United Kingdom [25–26],Germany [27], the United States [28], Brazil [29] and Australia [30] have shown that individuals with type 2 diabetes have a 1.5–2.5 fold higher risk of mortality compared to those without diabetes depending on the age of the patients studied.

Our results show excess mortality among underweight individuals (but not those who are overweight or obese) in comparison with individuals of normal weight in the overall study population, even after adjusting for age, gender, smoking and cancer. Previous studies and meta-analyses have shown a J or U-shaped relationship between BMI and mortality, such that both underweight and obesity are associated with excess mortality when compared to normal BMI [31–33]. While the paucity of individuals with higher grades of obesity in our study could have skewed our findings, it is also possible that being underweight confers a greater risk of mortality than expected in this population that is likely to have been exposed to chronic undernutrition and its attendant consequences such as infectious diseases and micronutrient deficiency in childhood and youth or even intrauterine growth retardation.

The present paper reports, for the first time, the relative contributions of various individual modifiable risk factors to all-cause mortality. Ischemic heart disease, which contributes 53% to the risk of mortality, is an eminently modifiable risk factor; it has been shown that more than 90% of all cases of acute myocardial infarction can be prevented by favorably modifying nine risk factors [34]. Diabetes, which is an equally significant contributor to the PAR, can also be prevented, or its onset significantly delayed, as has been shown by several large prospective studies [35]. Other important contributors to mortality such as physical inactivity, dyslipidemia, smoking and alcohol use and hypertension are also easily modifiable. Identification and modification of these risk factors will help reduce overall mortality and more importantly, prevent premature mortality in this population.

In the present study, excess mortality due to diabetes was highest in the age group of 51 to 70 years. This is possibly a reflection of the earlier age of onset of diabetes in this South Asian population, whereby patients develop diabetes in the fourth or fifth decade of life, and develop complications and are at risk of dying by the sixth or seventh decade. This has significant implications in that these age groups represent the apex of productive life in many occupations, and illness and death occurring at this time will therefore have a profound impact on the family, society and nation.

Renal failure contributed the most to excess mortality among individuals with diabetes, although it was overall cited as the second most common cause of death in this group, accounting for 10.7% of deaths. Globally, diabetes is the most important risk factor associated with death from renal failure among adults at all ages, and is now the most frequently occurring comorbidity among individuals with renal failure. Studies from different parts of the developed world have shown that declining renal function (indicated by increasing levels of albuminuria or by decreasing glomerular filtration rate) is a strong predictor of all-cause and cardiovascular mortality among individuals with diabetes [36–37]. Indeed, the NHANES data suggests that kidney disease is responsible for the excess mortality found among US adults with diabetes as compared to the general population [38]. Similarly, a study of 150,000 individuals from Mexico showed that renal disease accounted for the largest absolute excess of deaths due to diabetes [39].

CVD was also found to contribute significantly to excess mortality in individuals with diabetes. Globally, coronary artery disease is the leading cause of death and is predicted to remain so for the next 20 years [40–41]. Diabetes mellitus continues to be associated with an approximately 2 to 3 fold increased risk of CVD mortality [42], in spite of advances in diabetes management and preventive cardiology. There is a huge unmet need for therapeutic approaches to ensure global risk reduction so as to prevent premature death due to CVD in this population.

The strengths of our study are the representativeness of the sample and long duration of follow-up, use of standardized verbal autopsies to collect information on death from the community and a good response rate (82%). Ours is the first study to compare mortality rates among Asian Indians with and without diabetes, as also to evaluate the causes and characteristics of excess mortality in Asian Indians with diabetes, and the modifiable risk factors contributing to the PAR for death. The main limitation of the study is that we were unable to collect information on cause of death from those individuals who were ascertained to have died, but whose relatives were not traceable.

In conclusion, the age standardized mortality rate among urban Asian Indians in Chennai was 28.2 per 100, 000 population. Individuals with diabetes had three times higher mortality rates compared to those without. Renal disease, ischemic heart disease, and cerebrovascular disease accounted for most of the excess mortality due to diabetes which was most marked in the age group of 51–70 years. We have also shown that underweight (but not overweight or obesity) was associated with an increased risk of mortality in the overall study cohort. Favorably altering risk factors through increased physical activity, smoking cessation, good glycemic control, blood pressure control and lipid-lowering therapy, and preventing or delaying the onset of diabetes by means of lifestyle modification and judicious use of medications, could help to significantly reduce mortality and prevent premature deaths in this population.

## Supporting information

S1 Dataset CURES mortality dataset. (XLS)
